# Supplementary material for: Taxonomic and Phylogenetic Updates on Apiospora: Introducing Four New Species from Wurfbainia villosa and Grasses in China
Source: J Fungi (Basel). 2023 Nov 6;9(11):1087. doi: 10.3390/jof9111087 (PMC10671859; doi:10.3390/jof9111087)
Supplement: Supplementary file 1 [file jof-09-01087-s001.zip › jof-2592894-supplementary.pdf]

Supplementary Table S1. Synopsis of morphological characteristics of *Ap. marina* and its closely related species.

Characters	***Apiospora* species**				
	A. acutiapicum	***Ap. marina***	***Ap. paraphaeosperma***	***Ap. pseudorasikravindrae***	***Ap. rasikravindrae***
Host/substrate	*Bambusa bambos*/dead twigs	Seaweed	*Bambusa* sp./culms	*Bambusa dolichoclada*/sheath	Soil, *Coffea arabica*/leaf, *Pinus thunbergii*/wood, marine submerged wood, *Oryza granulate*/leaf
Conidiomata	350–450 mm, pycnidial, immersed, aggregated, scattered, subglobose, ostiolate, black, coriaceous	NA	NA	NA	NA
Conidiophores	Reduced to conidiogenous cells	NA	Reduced to conidiogenous cells	Basauxic, straight or flexuous, cylindrical, hyaline, thick, smooth-walled, aseptate, 10–15 × 3–7 mm (x = 12.3 × 5.2 µm)	Arising from swollen basal cells, micro-semi-macronematous, mononematous, straight or flexuous, unbranched, septate, smooth, thin-walled, hyaline to sub-hyaline, 5–90 × 1–1.5 µm
Conidiogenous cell	Erect, basauxic, cylindrical to ampulliform, apex pointed and hyaline, smooth and thick-	Aggregated in clusters on hyphae or solitary, hyaline, erect, ampulliform	Basauxic, aggregated in clusters on hyphae, hyaline, smooth, elongated, conical to ampulliform, 25–30 × 4–6	Holoblastic, ampulliform, cylindrical or doliiform, hyaline to olivaceous, 4–10 × 1.2–5 µm ($\bar{x}$ = 8.6 × 4.2 µm)	NA

	walled, pale brown, 4–7 × 2–3 µm		µm ($\overline{x}$ = 27 × 4.7 µm)		
Conidia	Globose in face view, subglobose to oval in the side view, brown to dark brown, smooth, apex and base blunted, dark equatorial slit, 7.5–10 × 8.5–12 µm	Brown, smooth to granular, globose to elongate ellipsoid, (9.5–)10–12 (− 13) × (7.5–)8.0–10 µm	Brown, smooth, somewhat granular, globose to ellipsoid in face view, with a median scar, lenticular in side view, with pale equatorial slit, 10–19 µm diam., ($\overline{x}$ = 13 µm)	Globose in face view, lenticular in side view, with a pale longitudinal slit, dark brown, thick-walled, finely roughened with one or two concentric pale rings, 5–10 × 5.5–11 µm ($\overline{x}$ = 9.3 × 10.1 µm)	Arising acropleurognously, dimorphous, lenticular, ovoid in face view, 10−15 × 6.0−10.5 µm; elongate to clavate conidia, 15−25 × 7.5−10 µm, smooth, double walled, brown to pale olivaceous with prominent truncate base and equatorial germ slit
Sterile cells	NA	NA	Forming on solitary loci on hyphae, brown, finely roughened, ellipsoid to clavate, 20–30 × 9–13 µm ($\overline{x}$ = 24 × 11 µm)	NA	NA
Reference	[28]	[52]	[52]	[28]	[60]

NA: not determined.

Supplementary Table S2. Morphospecies of *Apiospora.*

**Taxa**	**Strain Numbers**	**Substrates**	**Known lifestyle**	**Sexual/ Asexual morph**	**Countries**
Apiospora aberrans	-	On dead Bambusoideae (*Bambusa blumeana*)	Saprobe	Sexual	Philippines
Apiospora buddlejae	-	On branches of *Buddleja*	Saprobe	-	Portugal
Apiospora camarae	-	On twigs of *Osyris alba*	Saprobe	-	Portugal

Apiospora camptospora	Penzig 522	Dead leaves of *Saccharum officinarum*	Saprobe	Sexual	Jawa
Apiospora carpinea	-	-	Saprobe	Sexual	-
Apiospora chilensis	-	On stems of *Chusquea cumingii*	Saprobe	Sexual	Biobío, Los Lagos and Valparaíso
Apiospora comedens	-	On dead leaves	Saprobe	Sexual	Surinam
Apiospora curvispora	Balansa 3823	On rotten culms of Bambusaceae	Saprobe	Sexual	Paraguay
Apiospora deschampsiae	-	On leaf of *Deschampsia cespitosa*	Saprobe	Sexual	Switzerland
Apiospora imperatae	-	On *Imperata cylindrica*	Saprobe	Sexual	Philippines
Apiospora indica	Holotype HCIO 1229	On *Bambusa*	Saprobe	Sexual	Kerala
Apiospora inserta	-	On dead stems of arundinaceous plant	Saprobe	Sexual	Suriname
Apiospora liliacearum	-	On withered shoots of *Geitonoplesium cymosum* and *Smilax australis*	Saprobe	Sexual/ Asexual	New South Wales
Apiospora lonicerae	-	On *Lonicera maximowiczii* and *Lonicera coerulea*	-	-	former Soviet Union
Apiospora luzonensis	Merrill 3533	On dead culms of *Bambusa*	-	-	Philippines
Apiospora montagnei	-	On stems of *Arundinaria mauritanica*	Saprobe/ endophyt e	Sexual/ Asexual	Algeria, France
Apiospora muroiana	-	On dead culms of *Phyllostachys bambusoides*	-	-	Japan
Apiospora myrtincola	-	On branches of Myrtaceae	Saprobe	Sexual	São Paulo
Apiospora pachyspora	-	On living leaves of *Micania*	Saprobe	Sexual	Brazil
Apiospora parallela	-	In clum of *Calamagrostidis aridis*	Saprobe	Sexual	southern Finland
Apiospora petiolicola	-	On Tilia	Pathogen	Sexual	Germany
Apiospora phomatopsis	-	On rotten culms of *Zea mays*	Saprobe	Sexual	Buenos Aires
Apiospora phyllostachydicola	-	On dead culms of *Phyllostachys bambusoides*	Saprobe	None	Japan
Apiospora platensis	-	On rotten culms of *Pennisetum tristachyum*	Saprobe	Sexual	Buenos Aires
Apiospora punctum	-	On rotten leaves of *Molinia* and of *Dactylon*	Saprobe	Sexual	Italy
Apiospora rhododendri	03-1892	On dead branch of *Rhododendron*	Saprobe	Sexual	Netherlands
Apiospora rhodophila	-	On branches of *Rosa alpina*	Saprobe	Sexual	Italy
Apiospora rosae	-	*Rosa canina*	Saprobe	Sexual	Putten
Apiospora rosenvingei	-	On stem of *Betulae glandulosae*	Saprobe	Sexual	Narsak
Apiospora rottboelliae	Baker 917	On dead culms of *Rottboellia exaltata*	Saprobe	Sexual	Philippines
Apiospora rubi-fruticosi	-	On sarmens of *Rubus fruticosus*	Saprobe	Sexual	Italy

Apiospora rubi-ulmifolii	-	On twigs of *Rubus ulmifolius*	-	-	Morocco
Apiospora shiraiana	-	On culms of *Bambusa* sp.	-	-	Honshu
Apiospora siamicola	-	-	-	-	-
Apiospora sparsa	Baker 105	On dead culms of Poaceae	Saprobe	Sexual	Colombia
Apiospora sphaerosperma	-	-	-	-	-
Apiospora spinulosa	-	On dead *Panicum* sp.	Saprobe	Sexual	Hawaiian Is
Apiospora striola	-	in culm *Andropogonis, Phragmitis, Bambuseprope Parma*	Saprobe	Sexual	Italy
Apiospora tintinnabula	PDD 41022	On dead culms of *Bambusa*	Saprobe	Sexual/ Asexual	New Zealand
Apiospora urticae	-	On dry stems of *Urtica*	Saprobe	Sexual	Germany
Apiospora yerbae	-	On dead branches	Saprobe	Sexual	Misiones

Notes: "-" = information not available.
